# Nucleoside Analogs in ADAR Guide Strands Enable Editing at 5′-GA Sites

**DOI:** 10.3390/biom14101229

**Published:** 2024-09-29

**Authors:** Aashrita Manjunath, Jeff Cheng, Kristen B Campbell, Casey S. Jacobsen, Herra G. Mendoza, Leila Bierbaum, Victorio Jauregui-Matos, Erin E. Doherty, Andrew J. Fisher, Peter A. Beal

**Affiliations:** 1 Department of Chemistry, University of California, One Shields Avenue, Davis, CA 95616, USA; amanjunath@ucdavis.edu (A.M.); jefcheng@ucdavis.edu (J.C.); kcampbell@ucdavis.edu (K.B.C.); csjacobsen@ucdavis.edu (C.S.J.); hdgrajo@ucdavis.edu (H.G.M.); labierbaum@ucdavis.edu (L.B.); vijaureguim@ucdavis.edu (V.J.-M.); eedoherty@ucdavis.edu (E.E.D.); ajfisher@ucdavis.edu (A.J.F.); 2 Department of Molecular and Cellular Biology, University of California, Davis, CA 95616, USA

**Keywords:** ADAR, RNA editing, Nucleoside analog

## Abstract

Adenosine Deaminases Acting on RNA (ADARs) are members of a family of RNA editing enzymes that catalyze the conversion of adenosine into inosine in double-stranded RNA (dsRNA). ADARs’ selective activity on dsRNA presents the ability to correct mutations at the transcriptome level using guiding oligonucleotides. However, this approach is limited by ADARs’ preference for specific sequence contexts to achieve efficient editing. Substrates with a guanosine adjacent to the target adenosine in the 5′ direction (5′-GA) are edited less efficiently compared to substrates with any other canonical nucleotides at this position. Previous studies showed that a G/purine mismatch at this position results in more efficient editing than a canonical G/C pair. Herein, we investigate a series of modified oligonucleotides containing purine or size-expanded nucleoside analogs on guide strands opposite the 5′-G (−1 position). The results demonstrate that modified adenosine and inosine analogs enhance editing at 5′-GA sites. Additionally, the inclusion of a size-expanded cytidine analog at this position improves editing over a control guide bearing cytidine. High-resolution crystal structures of ADAR:/RNA substrate complexes reveal the manner by which both inosine and size-expanded cytidine are capable of activating editing at 5′-GA sites. Further modification of these altered guide sequences for metabolic stability in human cells demonstrates that the incorporation of specific purine analogs at the −1 position significantly improves editing at 5′-GA sites.

## 1. Introduction

RNA editing encompasses the insertion, deletion, or modification of nucleotides in RNA [1]. RNA base modifications can be catalyzed by several enzymes, and commonly occur in humans via the ADAR (Adenosine Deaminase Acting on RNA) family. There are three endogenous human ADARs, of which only ADAR1 and ADAR2 are catalytically active. These enzymes catalyze the hydrolytic deamination of adenosine (A) to inosine (I) in double-stranded RNA (dsRNA). Inosine pairs with cytidine (C) and can be read as guanosine (G), potentially altering translation, RNA secondary structure, and splicing [2,3,4]. In a process known as site-directed RNA editing (SDRE), chemically modified guide strands complementary to specific target sequences generate the requisite dsRNA that can recruit ADAR, facilitating A-to-I editing at predetermined locations in the targeted transcripts [5,6,7,8,9,10]. This can correct disease-causing mutations and restore wild-type functionality, or in some cases, create a new functional mutant with wild-type-like activity [11]. The delivery of a small oligonucleotide to recruit endogenous ADAR renders SDRE a promising therapeutic approach by eliminating the need to administer exogenous protein to facilitate editing. The efficacy with which ADARs can perform therapeutic base editing is dependent on the sequence context immediately surrounding the edited adenosine. ADARs prefer uridine (U) and guanosine (G) directly flanking the target A in the 5′ and 3′ directions, respectively. Previous studies have shown that substrates with a 5′-U have higher editing efficiencies when compared to substrates with other canonical bases at this position. While the 3′ base is important for editing, the 5′ position has a larger effect on editing efficiency [12].

**Figure 1:**
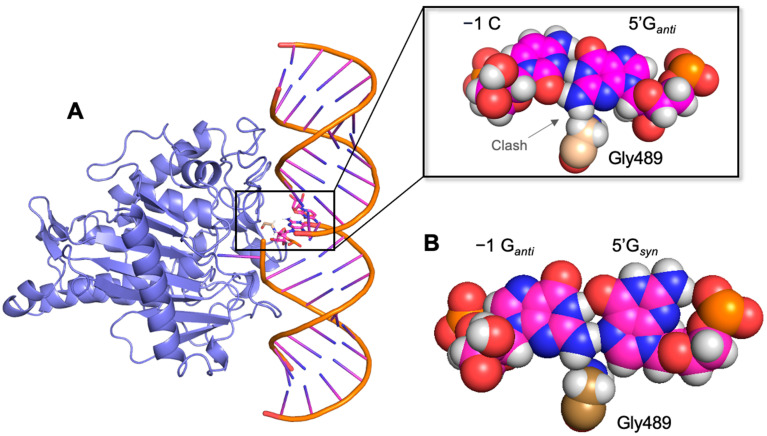
Steric clash with Gly489 relieved by G_syn_/G_anti_ pair. (**A**) Model of G/C pair in an ADAR2d/RNA complex suggests a steric clash with glycine residue 489 [13]. (**B**) A G/G pair where 5′-G is flipped to the syn conformation alleviates steric clash and enables editing at adjacent target adenosine.

Crystal structures of ADAR2 bound to dsRNA reveal key substrate–enzyme interactions that help explain the inefficient editing capabilities of ADAR for substrates with guanosine adjacent to a target adenosine in the 5′ direction (5′-G) [13]. Critical in the mechanism of the ADAR reaction, a loop in the protein (i.e., the base-flipping loop) approaches the site of reaction on the dsRNA from the minor groove and the side chain of a glutamic acid (Glu488) present on this loop flips the targeted A into the active site and fills the vacated space. Substrate RNAs with 5′-U are well accommodated while the 2-amino group on 5′-G clashes with Gly489, adjacent to Glu488 of the ADAR2 flipping loop (Figure 1A). Improving ADAR editing at sites containing a 5′-G nearest neighbor is important for expanding the scope of its use as a disease therapeutic. For instance, the common nonsense mutations in the *MECP2* gene linked to Rett syndrome could be repaired by ADARs [14]. These premature termination codons are converted by ADARs to tryptophan codons (UAG → UGG and UGA → UGG). However, UGA nonsense mutations pose a challenge to ADAR editing due to the presence of 5′-G adjacent to the target A. We previously demonstrated that diminished editing at such targets can be improved by inducing a conformational change at the 5′-G by pairing it with purine analogs at the −1 position of the guide oligonucleotide [15]. A guanosine paired with the 5′-G enables it to flip into the *syn* conformation, creating a stable G_syn_/G_anti_ pair. This pair is accommodated by ADAR2 adjacent to an editing site. While there is often no editing observed for sites bearing a 5′-GA target when 5′-G is paired with C on the guide strand, the conformational change afforded by the G_syn_/G_anti_ pair enables the 2-amino group to flip into the major groove of the RNA substrate. As a result, steric hinderance between the 2-amino group on the 5′-G and residue Gly489 in the ADAR2 flipping loop is alleviated, which enables proper enzyme function (Figure 1B). Structural studies of the RNA motif confirmed the *syn:anti* pairing interaction between a G/G mismatch, as well as a G/3-deaza-2′-deoxyadenosine (3-deaza dA) mismatch, leading us to propose that a purine:purine mismatch that flips 5′-G into the *syn* conformation is critical for enabling ADAR editing at this disfavored site. We extend these findings here by investigating the incorporation of several other purine analogs demonstrating editing at 5′-GA sites that match editing efficiencies in preferred ADAR substrate sequence contexts. We identify additional nucleoside analogs that accomplish this in vitro and in cellulo and report a proposed mechanism of action for these analogs based on crystal structures of a truncated homodimer of human ADAR2 bound to dsRNA substrates containing 2′-deoxyinosine or a base-expanded cytidine analog (dyC) paired to the 5′-G.

## 2. Materials and Methods

### 2.1. Target and Guide Oligonucleotide Synthesis

RNA target strands encompassing the human *MECP2* R255X and a modified mouse *IDUA* sequence were prepared as previously described [7]. All guide oligonucleotides bearing nucleoside analogs were synthesized on ABI 394 DNA synthesizer. 8-Azanebularine (8-azaN) phosphoramidite was purchased from Berry & Associates Inc. dyC phosphoramidite was synthesized according to protocols reported by Lee & Kool and Temburnikar et al. [16,17]. The phosphoramidite synthesis of 8-azainosine was completed using a revised protocol by Véliz et al. [18]. The revised synthesis can be found in Supplementary Scheme S1 followed by ^1^H and ^13^C NMR spectra. All other nucleoside phosphoramidites and ancillary reagents were purchased from Glen Research. Oligonucleotide synthesis proceeded at a 0.2 μmol scale on controlled-pore glass (CPG) columns. Upon completion, the columns were dried for 16 h under vacuum. Most oligonucleotides were cleaved from the solid support by treatment with 3:1 30% NH_4_OH/EtOH for 6–8 h at 55 °C. Oligonucleotides containing 8-Br dG were cleaved at room temperature for 24 h. The supernatant was evaporated to dryness. The resulting pellets containing 2′-OH protecting groups were treated with 250 μL of 1 M TBAF/THF for 24 h at room temperature. Oligonucleotide pellets containing 8-azanebularine were instead resuspended in 100 μL anhydrous DMSO and treated with 125 µL 55% TEA·3HF overnight at room temperature. Deprotected oligonucleotides were precipitated with 1 mL 1-butanol and 25 μL 3 M sodium acetate at −70 °C for >2 h. The solution was centrifuged at 13,200× *g* for 20 min, the supernatant was removed, and the pellet was resuspended in 500 μL nuclease-free H_2_O before purification as described below. All oligonucleotides were purified by denaturing polyacrylamide gel electrophoresis as previously described [6,7]. Guide oligonucleotide masses were confirmed by MALDI TOF mass spectrometry using a Bruker UltraFlextreme MALDI TOF/TOF mass spectrometer at the UC Davis Mass Spectrometry Facility (Davis, CA, USA). Molecular weights for editing oligonucleotide (EON)-bearing phosphorothioate backbone modifications were obtained from Novatia Inc. (Newton, PA, USA) using ESI-MS. Sequences and mass spectrometry data for all oligonucleotides are found in Supplementary Table S8.

### 2.2. Protein Purification

Full-length, wild-type human ADAR2 (ADAR2) with an N-terminal His_10_-tag was overexpressed in *S. cerevisiae* and purified as previously described [19,20,21]. Purified ADAR2 was stored in 20 mM Tris–HCl pH 8.0, 100 mM NaCl, 20% glycerol, and 1 mM BME at −70 °C. Human ADAR1 p110 with a C-terminal His_10_-tag was overexpressed in *S. cerevisiae* and purified as previously described [20].

### 2.3. ADAR Adenosine Deamination Assay

Purified RNA target and guide strands (refer to Supplementary Tables S1 and S2 for sequences) were resuspended in a hybridization buffer (100 nM target, 1000 nM guide, 1X TE, 100 mM NaCl) and allowed to anneal by heating to 95 °C for 5 min followed by a slow cooling to room temperature. Deamination kinetic analyses were performed under single turnover conditions. For ADAR2 reactions, 10 nM hybrids were incubated in a reaction solution of 15 mM Tris–HCl pH 7.5, 3% glycerol, 60 mM KCl, 1.5 mM EDTA, 0.003% NP-40, 3 mM MgCl_2_, 160 U/mL RNase inhibitor, and 1.0 μg/mL yeast tRNA for 15 min at 30 °C. Reactions were initiated by adding 100 nM ADAR2 (diluted and incubated for 15 min at 30 °C) to each reaction solution and allowed to proceed for 120 min. Aliquots (10 μL) of reaction solution were quenched in 190 μL 95 °C water at 1, 5, 10, 15, 30, 60, and 120 min. For human ADAR1 p110 reactions, 10 nM hybrids were incubated in a reaction solution of 15 mM Tris-HCl pH 7.5, 26 mM KCl, 40 mM potassium glutamate, 1.5 mM EDTA, 0.003% NP-40, 4% glycerol, 160 U/mL RNase inhibitor, and 1.0 μg/mL yeast tRNA for 15 min at 30 °C. Reactions were initiated by adding 100 nM ADAR1 p110 to each reaction solution and allowed to proceed as described above. Aliquots (5 μL) of each quenched sample were subjected to reverse transcription-PCR (RT-PCR) using an Access RT-PCR system (Promega, Madison, WI, USA) and purified by Zymo clean and concentrator PCR purification kit (Irvine, CA, USA) according to manufacturer protocol. Sanger sequencing of the resulting amplicons was carried out by Azenta Life Sciences (South San Francisco, CA, USA). Sequencing traces were analyzed for editing, using the ratio of G-to-A peak heights as a metric to calculate percent editing. Statistical analyses and nonlinear fits were conducted in Microsoft Excel 16 and GraphPad Prism 9.

### 2.4. Luciferase Reporter Assay for MECP2 R255X Editing

A 90-nucleotide sequence of the *MECP2* gene including the R255X mutation was ligated to a sequence encoding nanoluciferase (Promega, Madison, WI, USA), then subcloned into a pcDNA3.1 vector (Invitrogen, Waltham, MA, USA) using T4 DNA ligase (NEB, Ipswich, MA, USA) along with a sequenceencoding firefly luciferase (Promega, Madison, WI, USA). The sequence inserted into the pcDNA3.1 vector for this purpose is shown in Supplementary Table S5. There were no introns encoded downstream of the R255X mutation in this reporter so the premature termination codon was not expected to trigger nonsense mediated decay [22]. XL-10 Gold ultracompetent cells (Agilent, Santa Clara, CA, USA) were transformed with ligation products, and plasmids were isolated using a Promega PureYield Miniprep kit according to manufacturer protocol. Human ADAR2 was overexpressed using plasmids derived from the pcDNA3.1 vector (Invitrogen, Waltham, MA, USA) as previously described [23]. Human embryonic kidney 293T (HEK293T) cells were cultured in Dulbecco’s modified Eagle’s medium (DMEM), 10% fetal bovine serum, and 1% anti–anti at 37 °C and 5% CO_2_. Once cultivated cells reached 60–90% confluency, 2 × 10^4^ cells were seeded into 96-well plates. Cells were transfected at 24 h using Lipofectamine 3000 (ThermoFisher Scientific, Waltham, MA, USA). Transfection proceeded by incubating 50 ng dual luciferase plasmid, 150 ng ADAR2 plasmid, 0.3 μL P3000, and modified guide oligonucleotides with a solution of Lipofectamine 3000 transfection reagent in Opti-MEM Reduced Serum media (1.16 μL/well, ThermoFisher Scientific, Waltham, MA, USA). The transfection solutions were added to designated wells and incubated at 37 °C and 5% CO_2_. After 48 h, cells were lysed, and luciferase activity was initiated via the addition of reagents from a Nano-Glo Dual-Luciferase Reporter assay (Promega, Madison, WI, USA) to each well per manufacturer’s protocol. Quantified readouts from firefly and nanoluciferase luminescence were measured on a BMG Labtech plate reader. All statistical analyses were conducted in Microsoft Excel 16.

### 2.5. Analysis of in Cellulo Editing by Sanger Sequencing

The culture and transfection of HEK293T cells proceeded as described above. After 48 h, cells were lysed and total RNA was isolated using an RNAqueous Total RNA Isolation kit (ThermoFisher Scientific, Waltham, MA, USA). The isolated RNA was treated with ezDNAse (ThermoFisher, Waltham, MA, USA) at 37 °C for 10 min, then reverse-transcribed using a SuperScript IV VILO kit (ThermoFisher, Waltham, MA, USA) [24]. Samples were amplified by PCR with Phusion High-Fidelity DNA polymerase (NEB, Ipswich, MA, USA). PCR product was purified by 1% agarose gel and a QIAquick Gel Extraction kit (Qiagen, Venlo, The Netherlands). The DNA was sequenced by Azenta Life Sciences (Newtown, PA, USA). Sanger sequencing traces were analyzed for editing, using the ratio of G-to-A peak heights as a measure of percent editing.

### 2.6. Preparation of Duplex Structures for Crystallography

The modified hGLI1 32 nt RNA target strands were synthesized as described above (refer to Supplementary Table S6 for sequences) [15]. Separate RNA guide strands containing either 2′-deoxyinosine or dyC at the −1 position were also synthesized as described. As in previous structures, the edited strand contained the adenosine analog 8-azaN at the editing site [15]. Respective duplexes were hybridized in water in a 1:1 ratio by heating to 95 °C for 5 min and slow cooling to 30 °C.

### 2.7. Preparation of ADAR2 RD E488Q for Crystallography

Protein expression and purification were carried out by modifying a previously reported protocol [15,23]. *S. cerevisiae* BCY123 cells were transformed with a pSc-ADAR construct encoding ADAR2-R2D E488Q (corresponding to residues 214–701). Cells were streaked on yeast minimal media minus uracil (CM-ura) plates. A single colony was inoculated in a 15 mL CM-ura starter culture. After cultures were shaken at 300 rpm and 30 °C overnight, 10 mL of starter culture was used to inoculate each liter of yeast growth medium. After cells reached an OD600 between 1.0 and 2.0 (20–24 h), cells were induced with 110 mL of sterile 30% galactose per liter and protein was expressed for 6 h. Cells were collected by centrifugation at 5000× *g* for 10 min and stored at −70 °C. Cells were lysed in 750 mM NaCl in buffer A (20 mM Tris–HCl, pH 8.0, 5% glycerol, 35 mM imidazole, 1 mM BME, and 0.01% Triton X-100) with a microfluidizer. Cell lysate was clarified by centrifugation (39,000× *g* for 45 min). Lysate was passed over a 5 mL Ni-NTA column (Qiagen, Venlo, The Netherlands) equilibrated with buffer A with 750 mM NaCl, which was then washed in three steps with 50 mL of lysis buffer, wash I buffer (buffer A + 300 mM NaCl), and wash II buffer (buffer A + 100 mM NaCl + 35 mM imidazole). Protein was eluted with a 35–300 mM imidazole gradient in wash II buffer over 80 min at a flow rate of 1 mL/min. Fractions containing target protein were pooled and further purified on a 5 mL GE Healthcare Lifesciences Hi-Trap Heparin HP column in wash II buffer. The protein was washed with 50 mL of wash II buffer without BME and eluted with a 100–1000 mM NaCl gradient over 60 min at a flow rate of 1 mL/min. Fractions containing target proteins were pooled and cleaved with an optimized ratio of 1 mg of His-tagged TEV protease per 1 mg of protein. Cleavage was carried out for 4 h at room temperature without agitation, followed by overnight cleavage at 4 °C before the product was passed over another Ni-NTA column at a flow rate of 0.5 mL/min. Flowthrough was collected, dialyzed against 20 mM Tris, pH 8.0, 200 mM NaCl, 5% glycerol, and 1 mM BME, followed by concentration to just under 1 mL for gel filtration on a GE Healthcare (Chicago, IL, USA) HiLoad 16/600 Superdex 200 PG column. Fractions containing purified protein were pooled and concentrated to 4–6 mg/mL for crystallization trials.

### 2.8. Crystallization of Protein/RNA Complexes

Crystals of the ADAR2-R2D E488Q-hGLI1 (G/dI pair) RNA complex were grown at room temperature by the sitting-drop vapor-diffusion method. A solution of 1.0 μL volume containing 100 uM protein and 50 uM GLI1-dI RNA was mixed with 1.0 μL of 50 mM MOPS pH 7.0, 100 mM NaCl, and 9% PEG 4000. Crystals took approximately seven days to grow. A single crystal was soaked briefly in a solution of mother liquor plus 30% ethylene glycol before flash cooling in liquid nitrogen. Crystals of the ADAR2-R2D E488Q-hGLI1 (G/dyC pair) RNA complex were grown at room temperature by the sitting-drop vapor-diffusion method. A solution of 1.0 μL volume containing 100 uM protein and 50 uM GLI1-dyC RNA was mixed with 1.0 μL of 50 mM MOPS pH 7.0, 100 mM NaCl, and 10% PEG 4000. Crystals took approximately 20 days to grow. A single crystal was soaked briefly in a solution of mother liquor plus 30% ethylene glycol before flash cooling in liquid nitrogen.

### 2.9. X-ray Data Collection, Processing, and Refinement of the ADAR/dsRNA Complexes

X-ray diffraction data for both dI and dyC containing RNA substrates complexed with ADAR2-R2D were collected at Stanford Synchrotron Radiation Lightsource (SSRL) on crystals cooled to 100 K. Data for both crystals were processed using the autoPROC program [25], which employs XDS for integration [26]. Both crystals exhibited anisotropic diffraction patterns which extended beyond 2.7 Å resolution along the b* axis, but lower resolution along the a* and c* axes. Therefore, the software package of STARANISO was used for scaling data [27]. The structures were solved by molecular replacement using the previous ADAR2-R2D structure (PDBID/6vff), where both the 5′-G base and the corresponding −1 complement base were “truncated” to an abasic site to minimize phase bias in initial electron density calculations. The molecular graphics program COOT was used for model building for both structures [28]. The structures were refined using the PHENIX software package [29]. X-ray diffraction data processing and structure refinement statistics are listed in Table S7.

## 3. Results

**Figure 2:**
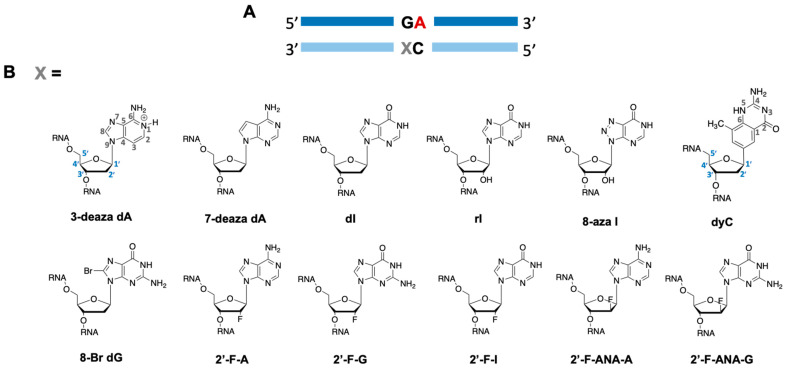
Analogs selected for testing at the −1 position of a guide oligonucleotide targeting a 5′-GA site. (**A**) Schematic of 5′-GA target editing sites wherein purine analogs are inserted across from 5′-G at the −1 position denoted as X. (**B**) Nucleoside analogs differing by base and sugar composition tested for their effect on ADAR-catalyzed deamination. Size-expanded cytidine analog (dyC) synthesis was published by Lee and Kool and synthesized in house for incorporation at −1 position [16,17]. Numbers on dyC and 3-deaza dA denote base and sugar numbering convention for dyC and all purine analogs, respectively.

**Figure 3:**
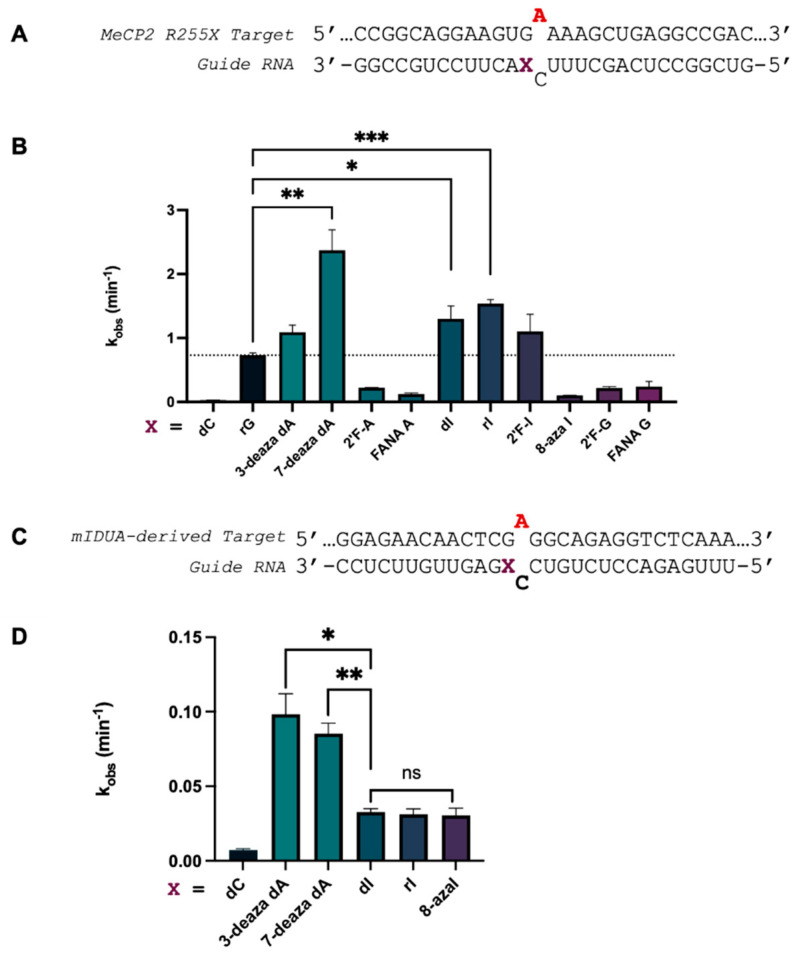
Effect of purine analogs at −1 position on deamination rate at two 5′-GA target sites. (**A**) Target RNA strand encoding MECP2 R255X mutation and guide oligonucleotide bearing various tested nucleoside analogs at −1 position denoted as X. Target adenosine is in red. (**B**) Observed rate constants for ADAR2 deamination of targets hybridized to guides containing each respective nucleoside analog. Reactions were carried out with 10 nM hybrid and 100 nM ADAR2. (**C**) Target RNA strand encoding a modified m*IDUA* mutation containing a 5′-GA site and guide oligonucleotide bearing various tested analogs at −1 position denoted as X. Bold indicates 2′-deoxynucleotide. Target adenosine is in red. Orphan cytidine is 2′-deoxyribonucleotide. (**D**) Observed rate constants for ADAR1 p110 deamination of targets hybridized to guides containing each respective analog. Reactions were carried out with 10 nM hybrid and 100 nM ADAR1 p110. Error bars, s.d. (*n* ≥ 3 technical replicates). A two-tailed Welch’s *t* test was conducted, where * *p* < 0.05, ** *p* < 0.01, *** *p* < 0.001.

*ADAR editing at 5′-GA sites is enhanced by purine analogs at the −1 position.* In an earlier report, we showed that ADAR substrates containing a guanosine adjacent to a target adenosine in the 5′ direction (5′-GA) could be efficiently edited provided that the 5′-G is paired with another purine on the guide strand that induced a *syn* conformation in the 5′-G. We sought to further contribute to the toolbox of ADAR-activating nucleotides at this position and match the editing efficiency shown in ADARs’ most preferred sequence contexts of 5′-U and 3′-G. The editing efficiency of a preferred target sequence has been shown to be heavily influenced by both the base and sugar modification pattern at the −1 position [6]. We selected ten different G, A, and I purine analogs based on their varied base and sugar modifications to determine if such a correlation exists in a disfavored target sequence (Figure 2A). Each of these nucleotides were placed opposite 5′-G at the −1 position in a guide strand targeting the methyl-CpG-binding protein 2 (MECP2) R255X mutation. The arginine to stop nonsense mutation results in a truncated MECP2 protein linked to Rett Syndrome. Upon ADAR editing at this site, restoration to wild-type does not occur; rather, a new sense codon is created (R255W) that allows for translation of a full-length protein. We subjected this target to ADAR editing by introducing ADAR2 to a duplex substrate containing the R255X sequence (Figure 3A) hybridized to our guide oligonucleotide bearing various −1 analogs. We found that each of the purine analogs tested increased ADAR2 editing efficiency compared to a G/dC pair at this position, while guides bearing 7-deaza-2′-deoxyadenosine (7-deaza dA), 2′-deoxyinosine (dI), and inosine (rI) enhanced editing beyond a G/G pairing and, along with the guide bearing 2′-fluroinosine (2′-F-I), edited at the same or higher rate as the G/3-deaza dA pairing (Figure 3B and Supplementary Table S3). We have shown in previous work that the G/3-deaza dA pair supported editing substantially better than a G/dA pair [15]. We further measured ADAR1 p110 editing rates with 7-deaza dA and inosine analogs in a duplex substrate derived from the mouse *IDUA* gene modified to bear a 5′-GA target sequence (Figure 3C). We found that the deazaadenosine analogs accelerated the rate of deamination more than the inosine analogs (Figure 3D and Supplementary Table S4). In contrast with results from the in vitro ADAR2 studies, all the inosine analogs exhibited the same deamination rate in ADAR1 p110, suggesting that their structural differences have little bearing on the overall rate-enhancing properties that inosine possesses.

**Figure 4:**
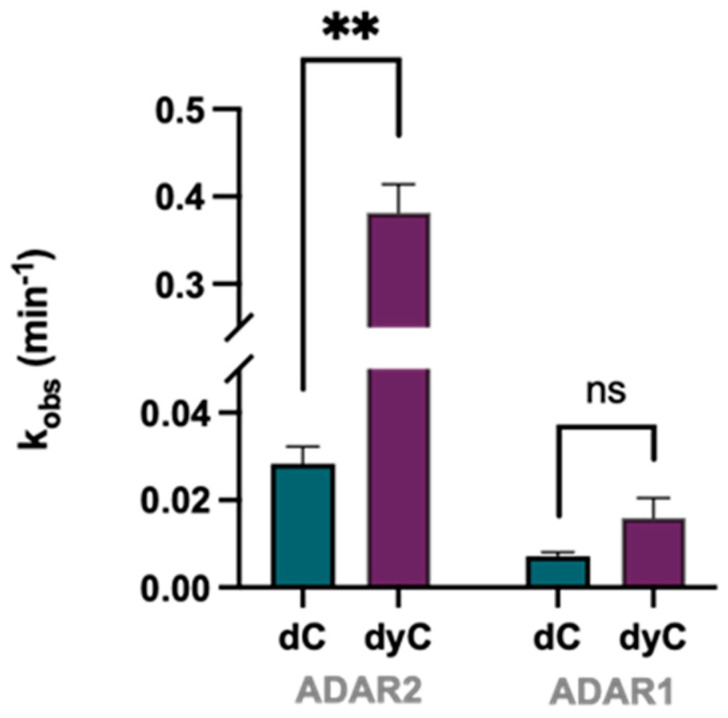
Effect of the dyC analog at the −1 position on deamination rate at two 5′-GA target sites in ADAR2 and ADAR1 p110. dyC and dC were inserted at the −1 position in guide strands as described in Figure 3A,C. Reactions were carried out with 10 nM hybrid and either 100 nM ADAR2 or 100 nM ADAR1 p110. Error bars, s.d. (*n* ≥ 3 technical replicates). A two-tailed Welch’s *t* test was conducted, where ** *p* < 0.01.

*Size expansion of a canonical nucleobase improves editing efficiency.* We were able to substantiate findings that a purine/purine mismatch directly upstream of a target adenosine with a 5′-G enhances ADAR editing rate, and structural studies indicate this is most likely due to their ability to induce the *syn* conformation in 5′-G [15]. However, we speculated that changing the size of the base pair involving the 5′-G might also serve to enable ADAR editing at these sites. Lee and Kool established that an expanded 2′-deoxycytidine bearing the same hydrogen bonding face as cytidine (dyC, Figure 2B) could be incorporated into oligonucleotides, but with a “widened” base structure via benzo-homologation between the base and sugar. The wider dyC/G base pair might create a minor groove structure that relieves the clash with Gly 489 and enable ADAR editing at the target A. Interestingly, the dyC-bearing guide targeting the *MECP2* R255X mutation shows a roughly 13-fold increase in ADAR2 deamination rate compared to the dC guide, while the advantage of the expanded base is diminished when used in reaction with ADAR1 p110 to deaminate the modified *mIDUA* transcript sequence (Figure 4). Rates of deamination for each of the dyC-modified guides remain lower than those bearing other purine analogs at the −1 position (e.g., dI, 7-deaza dA, etc.).

**Figure 5:**
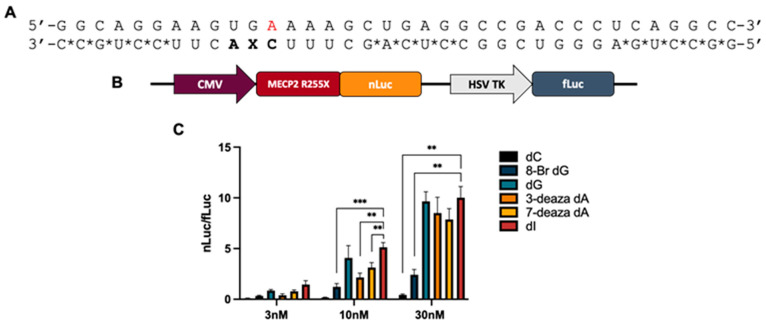
Dual luciferase assay measuring cellular editing with *MECP2* R255X reporter plasmid and overexpressed ADARs in HEK293 cells. (**A**) Guide oligonucleotide design for directed editing of the *MECP2* R255X in cellulo. Target A is highlighted in red. All nucleotides bear 2′-O-methyl modifications and contain phosphodiester linkages with the following exceptions: * indicates phosphorothioate modification, bold indicates 2′-deoxyribonucleotides. (**B**) Schematic of reporter plasmid construct encoding *MECP2* R255X mutation sequence followed by sequence encoding nanoluciferase and an independent promoter for firefly luciferase expression. (**C**) Relative nLuc/fLuc luminescence signals observed for guide oligonucleotides with varying identity of nucleoside analogs at −1 position and a 3 nM, 10 nM, and 30 nM concentration of guide with overexpressed ADAR2. Cells were lysed and luminesced at 48 h. A two-tailed Welch’s *t* test was conducted, where ** *p* < 0.01, *** *p* < 0.001.

*Guide strand modifications increase editing in cells when used in conjunction with metabolically stabilizing modifications*. To test the efficacy of our successful guide strands bearing −1 analogs in cells, we sought to measure directed editing using a dual luciferase assay [30]. For this purpose, we developed a luciferase reporter where repair of the premature termination codon created by the *MECP2* R255X mutation would allow for translation of a downstream nanoluciferase (nLuc). Independently, translation of a firefly luciferase (fLuc) sequence serves as a transfection control (Figure 5B). The ratio of nLuc/fLuc luminescence in this system is used as a proxy for editing efficiency. We and others have used similar luciferase assays to measure directed editing in cellulo [6,30]. The RNA guide design from our deamination kinetics experiments is incompatible with this cellular assay as those guides are susceptible to nuclease degradation. To mitigate this, we further modified the *MECP2* R255X guides bearing 7-deaza dA, 3-deaza dA, dG, dC, dI, and 8-bromo dG at the −1 position with a pattern of phosphorothioate backbone modifications. Additionally, we converted most of the 2′-OH RNA sugars in guides used in our kinetic studies to 2′-O-methyl sugars. Exceptions to this were the varying analogs of interest at the −1 position, flanked by two additional 2′-deoxynucleotides at the orphan and −2 positions (Figure 5A). We found that, upon transfection into HEK293T cells expressing ADAR2, guides bearing 3-deaza dA, 7-deaza dA, dI, and dG, all activated editing of the *MECP2* R255X target with 8-bromo dG substantially less efficient, particularly at the highest concentration tested (30 nM) (Figure 5C). Consistent with this observation, our earlier studies showed that 8-bromo dG at this position in a guide strand was not effective at enabling ADAR editing [7]. Weak editing activity was detected with the guide bearing dC at the −1 position. For comparison, we also directly measured editing efficiency for guides with dI and dC at the −1 position using RT-PCR and Sanger sequencing of the resulting amplicon (Supplementary Figure S1). Whereas editing with the dC guide was below that accurately measured by Sanger sequencing (<~5%), the dI guide supported 41 ± 10% editing for the MECP2 R255X target under these conditions.

**Figure 6:**
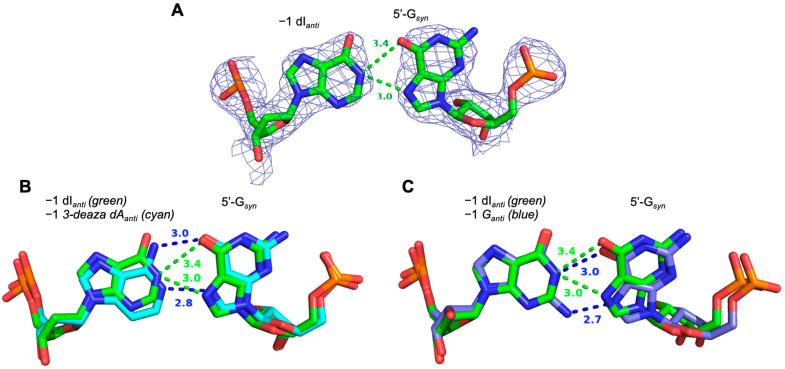
Structural analysis of purine analogs base paired with 5′-G in ADAR substrate RNA. (**A**) Fit of a G_syn_/dI_anti_ base pair in the 2*F_O_—F_C_* electron density map contoured at 1σ with hydrogen bond distances shown. (**B**) Superimposition of ADAR2 structures with RNA bearing 5′-G paired with 3-deaza dA (cyan) or 5′-G paired with dI (green). (**C**) Superimposition of ADAR2 structures with RNA bearing 5′-G paired with G (blue) or 5′ G paired with dI (green).

The X-ray crystal structure of an ADAR2 complex with RNA containing a dI/G pair shows the G adjacent to the target A adopts a *syn* conformation. Previously, we reported a high-resolution structure of an ADAR2-R2D-EQ/RNA complex showing the interaction between 5′-G and 3-deaza dA at the −1 position. This structure suggested 3-deaza dA donates two hydrogen bonds from its 6-amino and protonated N1 to the Hoogsteen face of the 5′-G [15]. We suggest that the 7-deaza dA studied here enables editing in a similar manner. However, since dI bears a single hydrogen bond donor on its Watson–Crick face, it must engage the 5′-G in a different manner than seen with 3-deaza dA. X-ray crystallography was used to resolve the structure of the ADAR2-R2D E488Q protein bound to an RNA duplex containing a target sequence derived from human glioma-associated oncogene 1 (hGLI1) and a guide oligonucleotide that forms a dI/G pair adjacent to the editing site. To trap the RNA/protein complex for crystallization, we synthesized a duplex substrate containing 8-azaN at the editing site [31]. We were able to grow crystals of the protein/RNA complex that diffracted X-rays to 2.8 Å (Figure 6A). The dI/G pair adjacent to the edit site is well defined and shows the 5′-G in the *syn* conformation with its Hoogsteen face accepting hydrogen bond donation from the protonated N1 of dI. The interaction appears to involve a bifurcated hydrogen bond with N1 of dI donating a strong hydrogen bond to N7 of 5′-G and a slightly weaker hydrogen bond to O6. Superimposition of the dI/G pair observed here with the previously published 3-deaza dA/G pair indicates that due to the bifurcated hydrogen bond, dI shifts in the direction of the major groove and away from the Gly489 position relative to the position of 3-deaza dA (Figure 6B). In addition, superimposition of the dI/G pair with the G/G pair clearly shows the benefit of removing the −1 G 2-amino in the minor groove in creating space for the flipping loop (Figure 6C).

**Figure 7:**
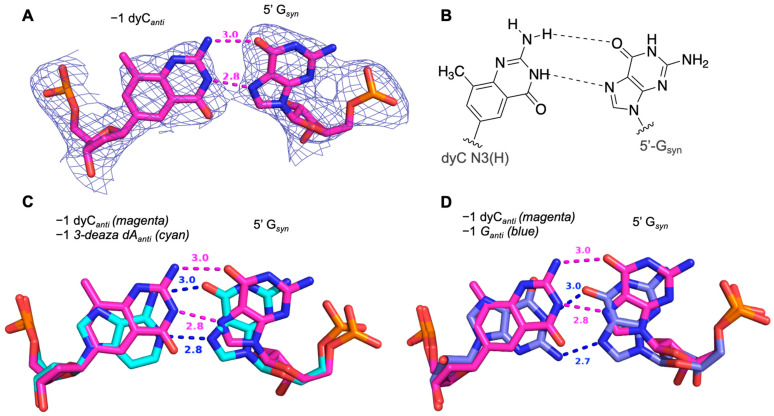
Structural analysis of an expanded cytidine analog base paired with 5′-G in ADAR substrate RNA. (**A**) Fit of a G_syn_/dyC base pair in the 2*F_O_—F_C_* electron density map contoured at 1σ with hydrogen bond distances shown. (**B**) Schematic of possible hydrogen bonding pattern for the N3. (H) dyC tautomer to Hoogsteen face of 5′-G_syn_. Superimposition of ADAR2 structures with RNA bearing 5′-G paired with 3-deaza dA (blue) or 5′-G paired with dyC (magenta). (**C**) Superimposition of ADAR2 structures with RNA bearing 5′-G paired with 3-deaza dA (cyan) or 5′-G paired with dyC (magenta). (**D**) Superimposition of ADAR2 structures with RNA bearing 5′-G paired with G (blue) or 5′-G paired with dyC (magenta).

The X-ray crystal structure of an ADAR2 complex with RNA containing a dyC/G pair shows the G adjacent to the target A adopts a *syn* conformation. We tested dyC at the −1 position because we imagined that a dyC/G pair that maintained the interaction of a canonical Watson–Crick G/C pair may create a minor groove edge that would allow the ADAR flipping loop to evade the steric clash between the 2-amino group on the G and residue Gly489. Since dyC improved ADAR2 editing efficiency compared to dC, we characterized further the nature of the dyC/G pair in an ADAR/RNA complex using X-ray crystallography. We formed a complex with ADAR2-R2D E488Q bound to an hGLI1 RNA substrate containing the dyC/G pair. Crystals of the complex diffracted X-rays to 2.7 Å and the structure was solved as described above. Interestingly, rather than an expanded Watson–Crick G/C-like pair, the expanded base induces the *syn* conformation at 5′-G and contacts its Hoogsteen face like the purines G, 3-deaza dA, and dI. A hydrogen bond of 3.0 Å is made between O6 and the 4-amino group of dyC (Figure 7A). The distance between N3 on dyC and N7 on 5′-G was measured at 2.8 Å, leading us to postulate that dyC may be in its N3(H) tautomer form with N3 donating a hydrogen bond to N7, further stabilizing the base-pairing interaction (Figure 7B) [32].

## 4. Discussion

Our previous work described the activation of the ADAR reaction at poorly edited substrates containing a guanosine adjacent to the target adenosine [15]. Therein, we showed that G and 3-deaza dA are suitable nucleosides to pair opposite the 5′-G by inducing a *syn* conformation and forming two hydrogen bonds on its Hoogsteen face. We show here that 7-deaza dA, several inosine analogs, and, to a lesser extent, the size-expanded dyC also facilitate editing at 5′-GA targets (compared to a C/G pair). Trends in editing efficiency across the tested analogs for the two different ADAR isoforms differed slightly, as shown by guides containing dyC or 8-azaI at the −1 position. The origins of these differences are not known at this time. Given that both 7-deaza dA and 3-deaza dA increase editing efficiency in 5′-GA substrates compared to canonical 2′-deoxy- or riboadenosine, we reasoned that ADAR activation via an adenosine analog paired with 5′-G is at least in part *p*Ka-dependent. Both 7- and 3-deaza dA have a higher N1 *p*Ka than adenosine, rendering a higher percentage of protonation at that site under ADAR reaction conditions (pH 7.5). Thus, we suggest 7-deaza dA forms two hydrogen bonds on the Hoogsteen face of the 5′-G in a manner similar to that seen with 3-deaza dA. However, how inosine analogs or dyC might interact with 5′-G in ADAR/RNA complexes was not as clear. Interestingly, we found that both nucleosides induce the 5′-G into the *syn* conformation and interact with its Hoogsteen face. The crystal structure bearing a dI/G pair suggests a bifurcated hydrogen bond involving the N1 of dI with distances of 3.4 Å to O6 and 3.0 Å to N7 of the 5′-G. This interaction causes the dI base to slide in the direction of the major groove, a finding that, in combination with the elimination of the 2-amino group as seen in the −1 G, creates space in the minor groove to better accommodate the flipping loop compared to both 3-deaza dA and G at the −1 position. We hypothesize that despite the favorable structural benefits offered by dI at the −1 position, bifurcation of one hydrogen bond may be less stabilizing to the *syn:anti* pair than the two hydrogen bonds present in the deaza-dA/G substrates. This may explain the lower in vitro deamination rates on substrates containing a −1 dI. The expanded C base dyC induces the 5′-G to flip *syn* and hydrogen bonds to the Hoogsteen face. We suggest dyC forms a base pair with the 5′-G in this conformation stabilized by two hydrogen bonds, involving the N3(H) tautomeric form of dyC. There is evidence that the related cytidine analog pseudoisocytidine can exist in its N3(H) tautomeric form [33]. Apart from dyC’s benzo-homologated motif, it bears structural similarity to pseudoisocytidine, leading us to believe that the N3(H) tautomer can form and donate a hydrogen bond to N7 on 5′-G, though we do not yet know the abundance of this tautomer under ADAR reaction conditions. It remains unclear as to why dyC does not enable editing as efficiently as the other purine analogs, as the dyC/G pair also slides in the direction of the major groove to alleviate steric clash with Gly489. Additionally, the backbone of the dyC/G pair closely matches the analogous G/G and 3-deaza dA/G pairs from our previously published structure (Figure 7C,D). The propensity for dyC to tautomerize under our deamination reaction conditions may also limit its ability to form a stable pair with the 5′ G.

Finally, our dual luciferase assay results confirm our biochemical finding that while C pairing with the 5′-G is inhibitory to editing, dG, 3-deaza dA, 7-deaza dA, and dI can all be incorporated opposite the 5′-G and, in combination with metabolically stabilizing guide oligonucleotide modifications, induce ADAR editing at these sites in cellulo. Importantly, simply forming a purine/purine mismatch at this position is insufficient as 8-bromo dG was shown to be ineffective at supporting ADAR editing in the dual luciferase assay. 8-Bromo dG is forced to adopt a *syn* conformation itself due to the bromine atom present at the 8-position, rendering it unavailable to create a stable hydrogen bonded base pair with the Hoogsteen face of the 5′-G [34].

## 5. Conclusions

We conclude that the critical features for a nucleoside analog to support editing at 5′-GA sites are the following: (1) an ability to stably interact with the Hoogsteen face of the 5′-G in a *syn* conformation and (2) the formation of a base pair with the 5′-G where the minor groove edge structure accommodates proper positioning of the ADAR flipping loop.

## Data Availability

Atomic coordinates and the structure factors have been deposited in the Protein Data Bank (PDB, https://www.rcsb.org/) with the following accession codes: 9d5j for ADAR2-R2D complexed with dsRNA containing deoxyinosine at the −1 position, and 9d5k for ADAR2-R2D complexed with dsRNA containing the base-expanded cytidine analog at the −1 position.

## Supplementary Materials

The following supporting information can be downloaded at: https://www.mdpi.com/article/10.3390/biom14101229/s1. Ref. [35].
