# Supplementary material for: Nucleoside Analogs in ADAR Guide Strands Enable Editing at 5′-GA Sites
Source: Biomolecules. 2024 Sep 29;14(10):1229. doi: 10.3390/biom14101229 (PMC11506087; doi:10.3390/biom14101229)
Supplement: Supplementary file 1 [file biomolecules-14-01229-s001.zip › biomolecules-3193092-supplementary.pdf]

Supporting Information

Nucleoside Analogs in ADAR Guide Strands Enable Editing at 5'-GA Sites

Aashrita Manjunath[1], Jeff Cheng[1], Kristen B Campbell[1], Casey S. Jacobsen[1], Herra G. Mendoza[1], Leila Bierbaum[1], Victorio Jauregui-Matos[1], Erin E. Doherty[1], Andrew J. Fisher[1,2] and Peter A. Beal[1]*

[1]Department of Chemistry, University of California, One Shields Avenue, Davis, CA 95616, United States

[2]Department of Molecular and Cellular Biology, University of California, One Shields Avenue, Davis, CA 95616, United States

Contents

Table S1 Guide sequences for *in vitro* deamination of the *MECP2* R255X target 3

Table S2 Guide sequences for *in vitro* deamination of the modified *IDUA* target 3

Table S3 Rate constants for *in vitro* deamination of the *MECP2* R255X target 2

Table S4 Rate constants for *in vitro* deamination of the modified *IDUA* target 2

Table S5 Sequences for *in cellula* editing of the *MECP2* R255X target 4

Table S6 Oligonucleotide sequences for crystallography. 4

Table S7 Data processing and refinement statistics for ADAR2-R2D:dsRNA complexes 4-5

Table S8 Oligonucleotide mass spectrometry data 6

Scheme S1 Modified synthesis of 8-azainosine from 8-azaadenosine..7-9

Figure S1 Quantification of *in cellula* percent editing by Sanger sequencing................................10

Table S1 Sequences for *in vitro* deamination of the *MECP2* R255X target [1]. Target adenosine is highlighted in red. All PCR primers are 2'-deoxynucleotides. All guides are ribonucleotides. [X] denotes varied nucleobases at this position. [X] encompasses dC, dG, 3-deaza-2'-deoxyadenosine, 7-deaza-2'-deoxyadenosine, 2'-fluoroadenosine, 2'-fluoro-arabinoadenosine, 2'-fluoroguanosine, 2'-fluoro-arabinoguanosine, 2'-fluroinosine, riboinosine, 2'-deoxyinosine, 8-azainosine, and dyC.

Human *MECP2* R255X target DNA template sequence (target A indicated in red, T7 promoter highlighted in grey)	CACGATTAATACGACTCACTATAGGGTGTGCAGGTGAAAAGGTCCTGGAG AAAAGTCCTGGGAAGCTCCTTGTCAAGATGCCTTTTCAAACTTCGCCAGGGG GCAAGGCTGAGGGGGGTGGGGCCACCACATCCACCCAGGTCATGGTGATC AAACGCCCCGGCAGGAAGTGAAAAGCTGAGGCCGACCCTCAGGCCATTCCC AAGAAACGGGGCCGAAAGCCGGGGAGTGTGGTGGCAGCCGCTGCCGCCG AGGCCAAAAAGAAAGCCGTGAAGGAGTCTTCTATCCGATCTGTGCAGGAGA CCGTACTCCCCATCAAGAA
MECP2 R255X guide strands	5'-GUCGGCCUCAGCUUUC[X]ACUUCCUGCCGG -3'
MECP2 R255X RT-PCR forward and sequencing primer	5'-GGGTGTGCAGGTGAAAAGG-3'
MECP2 R255X RT-PCR reverse primer	5'-TCTTGATGGGGAGTACGGTC-3'

Table S2 Sequences for *in vitro* deamination of the sequence-modified *IDUA* target [1]. Target adenosine is highlighted in red. All PCR primers are 2'-deoxynucleotides. All guides are ribonucleotides except for the following: bold indicates 2'-deoxynucleotide. [X] denotes varied nucleobases including 2'-deoxycytidine, dyC, 3-deaza-2'-deoxyadenosine, 7-deaza-2'-depxyadenosine, I, deoxyinosine, and 8-azainosine.

Mouse IDUA target with 5' G DNA template sequence (target A indicated in red, T7 promoter highlighted in grey)	TAATACGACTCACTATAGGGCTCCTCCCATCCTGTGGGCTGAACAGTAT AACAGACTCCCAGTATACAAATGGTGGGAGCTAGATATTAGGGTAGGA AGCCAGATGCTAGGTATGAGAGAGCCAACAGCCTCAGCCCTCTGCTTG GCTTATAGATGGAGAACAACTCGAGGCAGAGGTCTCAAAGGCTGGGG CTGTGTTGGACAGCAATCATACAGTGGGTGTCCTGGCCAGCACCCATCA CCCTGAAGGCTCCGCAGCGGCCTGGAGTACCACAGTCCTCATCTACACT AGTGATGACACCCACGCACACCCCGGATCC
IDUA guide strands	5'-UUUGAGACCUCUGUC**C**[X]GAGUUGUUCUCC-3'
Idua RT-PCR forward and sequencing primer	5'-GCTCCTCCCATCCTGTGGGCTGAACAGT-3'
Idua RT-PCR reverse primer	5'-CGGGGTGTGCGTGGGTGTCATCACT-3'

Table S3. Rate constants for ADAR2 *in vitro* deamination of *MECP2* R255X substrate with varying -1 gRNA modifications at the -1 position.[a]

-1 modification [a]	k_{obs} (min^{-1})[b]	k_{rel}[c]
dC	0.028 ± 0.003	1
dyC	0.38 ± 0.03	13
rG	0.73 ± 0.03	26
2'-F-G	0.22 ± 0.02	7.7
2'-F-ANA-G	0.24 ± 0.08	8.5
2'-F-A	0.223 ± 0.002	7.9
2'-F-ANA-A	0.12 ± 0.02	4.2
3-deaza dA	1.1 ± 0.1	39
7-deaza dA	2.4 ± 0.3	85
dI	1.3 ± 0.2	46
I	1.54 ± 0.06	54
8-azaI	0.103 ± 0.003	3.6
2'-F-I	1.1 ± 0.3	39

[a] Reactions were carried out with 10 nM of target RNA and 100 nM ADAR2 WT
[b] Reactions were fitted to the equation $[P]_t = \alpha[1 - \exp(-k_{obs} \cdot t)]$
[c] $k_{rel} = k_{obs}$ for modification/k_{obs} for deoxycytidine

Table S4. Rate constants for ADAR1 *in vitro* deamination of the sequence-modified *IDUA* substrate with varying -1 gRNA modifications at the -1 position.[a]

-1 modification [a]	k_{obs} (min^{-1})[b]	k_{rel}[c]
dC	0.0072 ± 0.0009	1
dyC	0.016 ± 0.005	2.2
3-deaza dA	0.10 ± 0.01	14
7-deaza dA	0.085 ± 0.007	12
dI	0.033 ± 0.002	4.6
rI	0.031 ± 0.004	4.3
8-azaI	0.030 ± 0.005	4.2

[a] Reactions were carried out with 10 nM of target RNA and 100 nM ADAR1 p110
[b] Reactions were fitted to the equation $[P]_t = \alpha[1 - \exp(-k_{obs} \cdot t)]$
[c] $k_{rel} = k_{obs}$ for modification/k_{obs} for deoxycytidine

Table S5 Sequences for *in cellula* editing experiments. Target adenosine is highlighted in red. For editing oligonucleotides (EONs), all nucleotides are 2'-O-methyl except for the following: **bold** indicates 2'-deoxynucleotides, * indicates a phosphorothioate linkage, and [X] denotes varied nucleobases at this position including dC, dG, 3-deaza-2'-deoxyadenosine, 7-deaza-2'-deoxyadenosine, deoxyinosine, and 8-bromo-2'-deoxyguanosine. All plasmid inserts and PCR primers 2'-deoxynucleotides.

Human *MECP2 R255X* sequence for dual luciferase assay reporter plasmid (target A indicated in red)	5'-ACCACATCCACCCAGGTCATGGTGATCAAACGCCCCGGCAGGAAGTGAAAAGCTGAGGCCGACCCTCAGGCCATTCCCAAGAAACGGGGC-3'
RT-PCR forward primer for sequencing of cellular editing	5'-AGGAGGACCTGGAATTCA-3'
RT-PCR reverse primer for sequencing of cellular editing	5'-GGACACACCTCCCTGTTCAA-3'
Human *MeCP2 R255X* cellular guides	5'-G*G*C*C*U*G*AGGGUCGGC*C*U*C*A*GCUUU**C**[X]**A**CUU*C*C*U*G*C*C-3'

Table S6 Sequences for crystallography. All nucleotides are ribonucleotides [2]. (N) is 8-azanebularine. (dI) is deoxyinosine, dyC is a widened cytidine analog [3].

GLI1(GG, G3dA) 32mer top with 8-azanebularine	5'- GCUCGCGAUGCG(N)GAGGGCUCUGAUAGCUACG -3'
GLI1 (dI) 32mer bottom	5'- CGUAGCUAUCAGAGCCCCCC(dI)GCAUCGCGAGC -3'
GLI1 (dyC) 32mer bottom	5'- CGUAGCUAUCAGAGCCCCCC(dyC)GCAUCGCGAGC -3'

Table S7 Data processing and refinement statistics for ADAR2-R2D bound to dsRNA substrates.

dsRNA Substrate	GLI1 G:dyC w/azaN 32mer	GLI1 G:dI w/azaN 32mer
PDBID	9D5K	9D5J
Synchrotron (Beamline)	SSRL (12-1)	SSRL (12-1)
Wavelength (Å)	0.97946	0.97946
Space Group	*C*2	*C*2
Unit Cell Parameters	a = 174.77Å, b = 63.11Å, c = 141.06Å, β = 118.54°	a = 174.69Å, b = 63.13Å, c = 141.78Å, β = 118.23°
Resolution Range (Å)	62 – 2.70 (2.88 – 2.70)	50 – 2.80 (3.08 – 2.80)
No. observed reflections	96,634 (4,616)	77,847 (3,902)
No. unique reflections	27,371 (1,369)	21,944 (1,097)
Ellipsoid Completeness[a]	92.0% (64.4%)	89.9% (48.6%)
Spherical Completeness[a]	72.7% (20.6%)	65.0% (13.4%)
I/σ (I)	10.3 (2.1)	7.4 (1.5)
R_{merge}[b] (%)	7.0 (62.3)	9.8 (85.2)
$CC_{1/2}$	0.897 (0.704)	0.998 (0.602)
**Refinement Statistics**		
R_{factor}[c] (%)	22.09	18.8
R_{free}[c] (%)	26.83	23.47
RMS bond length (Å)	0.013	0.009
RMS bond angle (°)	1.635	1.137
**Ramachandran Plot Statistics[d]**		
Favored (%)	93.77	90.26
Allowed (%)	5.99	9.25
Outliers (%)	0.24	0.48
**No. of atoms**		
Protein	6,482	6,559
RNA	1,362	1,359
Inositol Hexakisphosphate (IHP)	72	72
Zn	2	2
Mg	1	0
Waters	34	15
Ethylene Glycol	0	12

[a] Anisotropic data processing using autoPROC [4] and STARANISO [5] software package.

[b] $R_{merge} = [\sum_h \sum_i |\mathbf{I}_h - \mathbf{I}_{hi}| / \sum_h \sum_i \mathbf{I}_{hi}]$ where $\mathbf{I}_h$ is the mean of $\mathbf{I}_{hi}$ observations of reflection h. Numbers in parenthesis represent highest resolution shell.

[c] R-Factor and [c] Rfree = $\sum ||F_{obs}| - |F_{calc}|| / \sum |F_{obs}|$ x 100 for 95% of recorded data (R-Factor) or 5% data (Rfree).

[d] Ramachandran plot statistics from MolProbity [6].

Table S8 Oligonucleotide mass spectrometry data.

Oligo	Calculated Mass (Da)	Observed Mass (Da)
MECP2 R255X -1 3-deaza dA	9139	9134
MECP2 R255X -1 7-deaza dA	9124	9127
MECP2 R255X -1 2'-F-A	9149	9146
MECP2 R255X -1 2’-FANA-A	9149	9146
MECP2 R255X -1 dI	9130	9129
MECP2 R255X -1 rI	9152	9145
MECP2 R255X -1 2'-F-I	9154	9147
MECP2 R255X -1 2'-F-G	9164	9162
MECP2 R255X -1 dyC	9172	9173
MECP2 R255X -1 rG	9169	9164
MECP2 R255X -1 dC	9108	9108
MECP2 R255X -1 2’F-ANA-G	9176	9175
MECP2 R255X -1 8-azaI	9179	9178
IDUA -1 3-deaza dA	9090	9086
IDUA -1 8-azaI	9123	9121
IDUA -1 7-deaza dA	9081	9086
IDUA -1 dI	9085	9091
IDUA -1 rI	9114	9107
IDUA -1 dyC	9142	9139
IDUA -1 dC	9075	9080
EON R255X -1 dC	12094	12094
EON R255X -1 dG	12134	12134
EON R255X -1 7-deaza dA	12117	12117
EON R255X -1 3-deaza dA	12117	12117
EON R255X -1 dI	12119	12119
EON R255X -1 8-Bromo dG	12212	12212
Gli1 8-aza top strand	10321	10328
Gli1 dI bottom strand	10177	10176
Gli1 dyC bottom strand	10215	10215

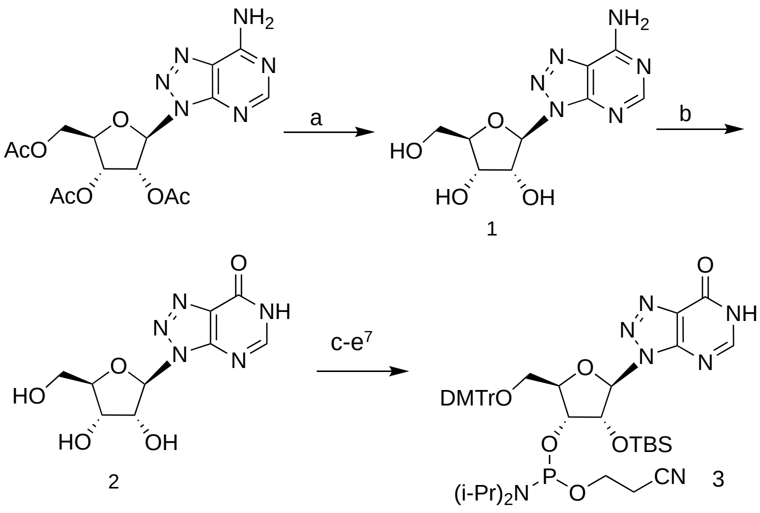

Scheme 1. Synthesis of 8-azainosine from 8-azaadenosine. (a) 7N NH_3/MeOH, 25 °C, 95%; (b) Adenosine deaminase, 200 U/mg, 0.1 M Na_3PO_4, 85%; (c-e) as previously published [7].

Synthetic procedures.

8-Azaadenosine (1). 2′,3′,5′-Tri-*O*-acetyl-8-azaadenosine (2.3 g, 5.7 mmol) was dissolved in 27 mL of methanolic ammonia 7N. The mixture was sealed and stirred for 16 h at room temperature. The resulting white solid was resuspended in dichloromethane and concentrated under reduced pressure. The resulting solid was then resuspended in *n*-hexanes, filtered, and washed with 30 mL of a mixture of 3:1 Ethyl acetate:*n*-hexanes and 30 mL of 1% MeOH in dichloromethane. The resulting filter cake was dried down under reduced pressure to afford 8-azaadenosine (1.44g, 95%) as a white solid and used without further purification. ^{1}H NMR (300 MHz, DMSO) δ 8.55 (s, 1H), 8.32 (s, 1H), 8.21 (s, 1H), 6.16 (d, *J* = 5.2 Hz, 1H), 5.58 (d, *J* = 6.0 Hz, 1H), 5.30 (d, *J* = 5.3 Hz, 1H), 5.03 (dd, *J* = 6.7, 5.2 Hz, 1H), 4.86 (q, *J* = 5.3 Hz, 1H), 4.30 (q, *J* = 4.8 Hz, 1H), 4.01 (p, *J* = 5.0 Hz, 1H), 3.68 – 3.60 (m, 1H), 3.56 – 3.45 (m, 1H).^{13}C NMR (76 MHz, DMSO) δ 157.41, 156.74, 149.33, 124.65, 90.23, 86.75, 73.42, 71.26, 62.42. HRMS calc. [M-H]: 267.08, obsd. [M-H]: 267.0840

8-Azainosine (2). ADA (368 µL ; Sigma, 200 units/mg protein, calf intestinal mucosa from a 2 mL stock) was added to a suspension of 8-azaadenosine (369 mg, 1.38 mmol) in aqueous sodium phosphate buffer (0.1 M, 33 mL; pH 7.4) and stirring was continued at room temperature overnight. The reaction mixture was concentrated under reduced pressure and the residue was purified by flash column chromatography (10-20% MeOH/$CHCl_3$). The chromatographed solid was then washed and filtered with 10 mL of a mixture of H_2O:MeOH:EtOH (0.5:5.5:4) at 0 °C to afford 8-azainsoine (295 mg, 80%) as a white solid. ^{1}H NMR (400 MHz, DMSO) δ 12.83 (s, 1H), 8.31 (s, 1H), 6.11 (d, *J* = 4.7 Hz, 1H), 5.63 (d, *J* = 5.9 Hz, 1H), 5.31 (d, *J* = 5.5 Hz, 1H), 4.79 (dt, *J* = 17.6, 5.3 Hz, 2H), 4.30 (q, *J* = 4.7 Hz, 1H), 4.03 – 3.99 (m, 1H), 3.61 (dt, *J* = 11.8, 4.8 Hz, 1H), 3.48 (dt, *J* = 11.7, 5.7 Hz, 1H).^{13}C NMR (76 MHz, MeOD:H_2O 3:1) δ 156.59, 149.97, 149.09, 129.94, 90.21, 86.05, 74.13, 70.96, 61.90, 47.93. HRMS calc, [M-H]: 268.07, obsd. [M-H]: 268.0680

^{1}H NMR (300 MHz, DMSO) 8-azaadenosine

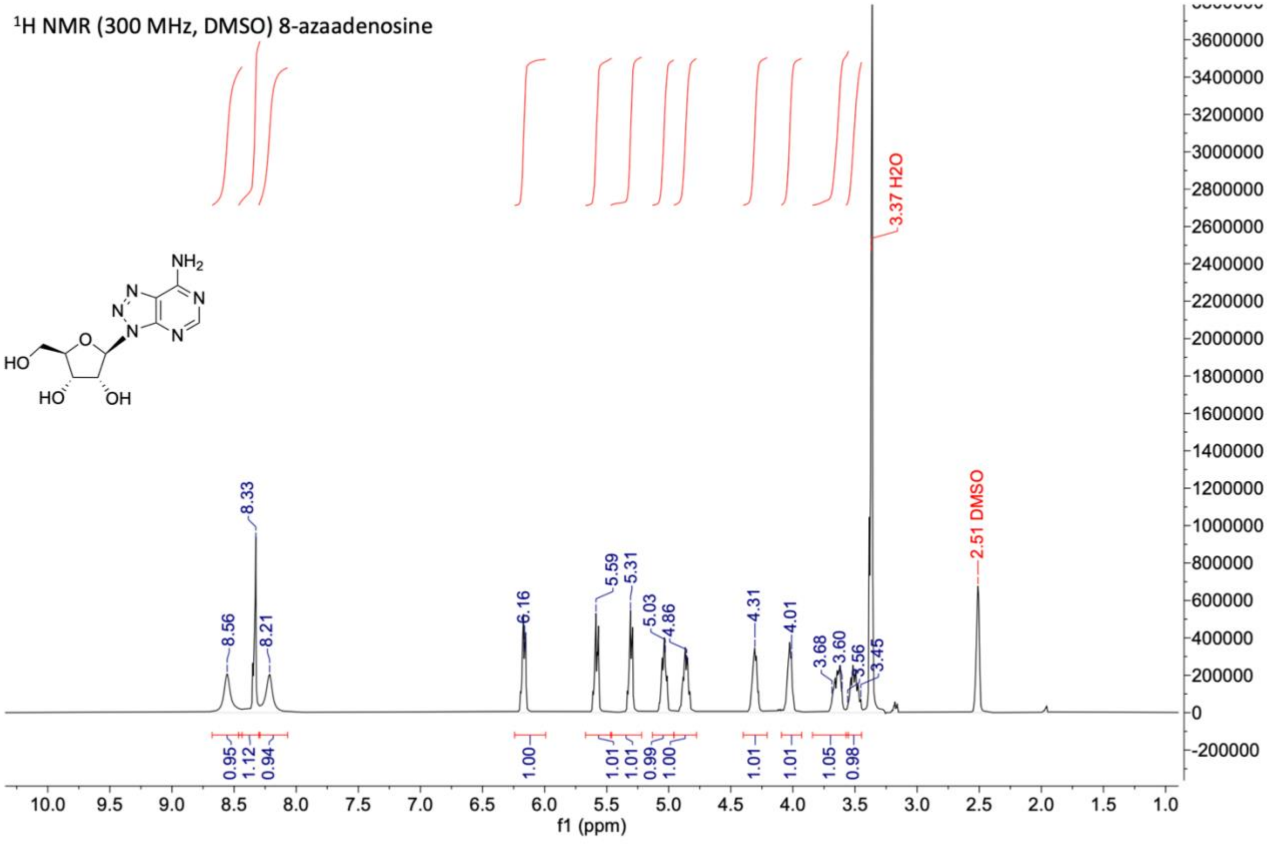

^{13}C NMR (76 MHz, DMSO) 8-azaadenosine

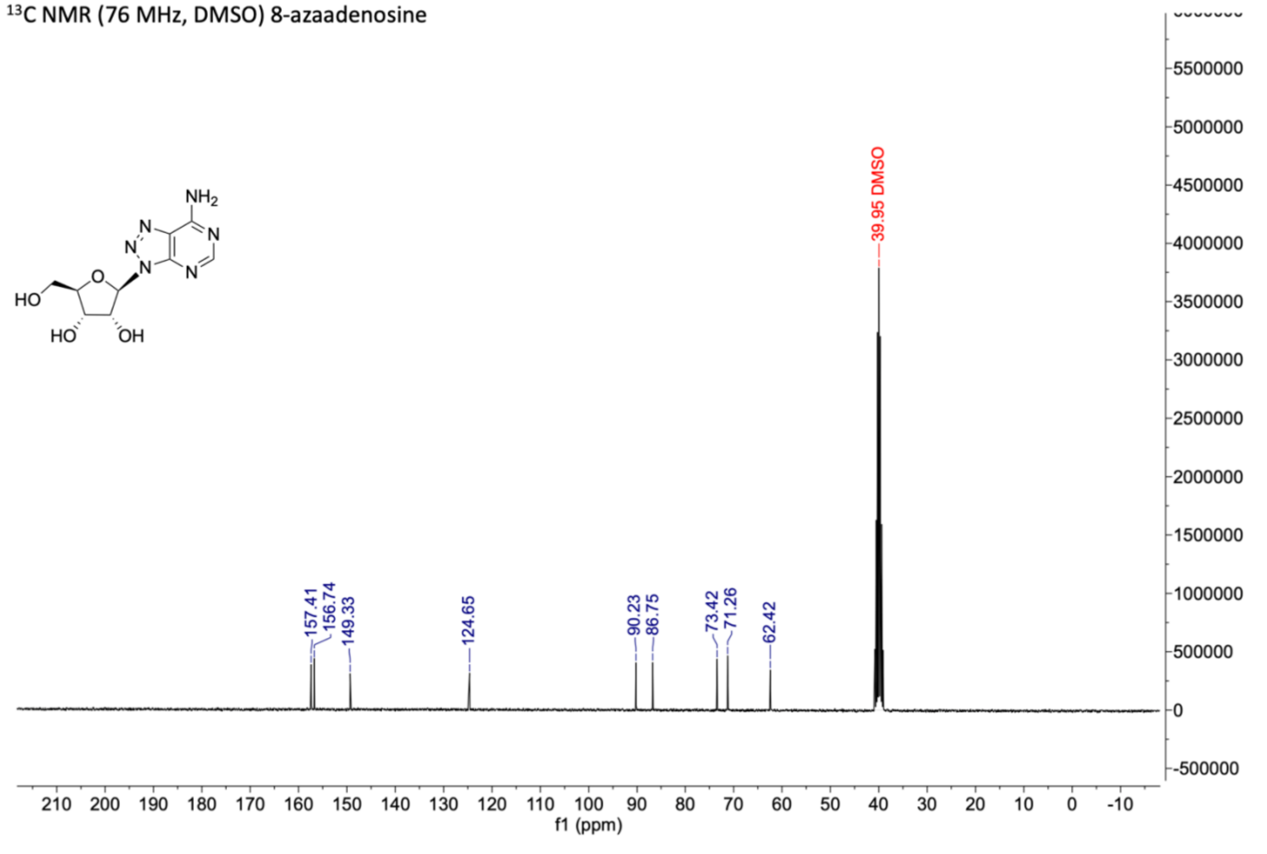

^{1}H NMR (300 MHz, DMSO) 8-azainosine

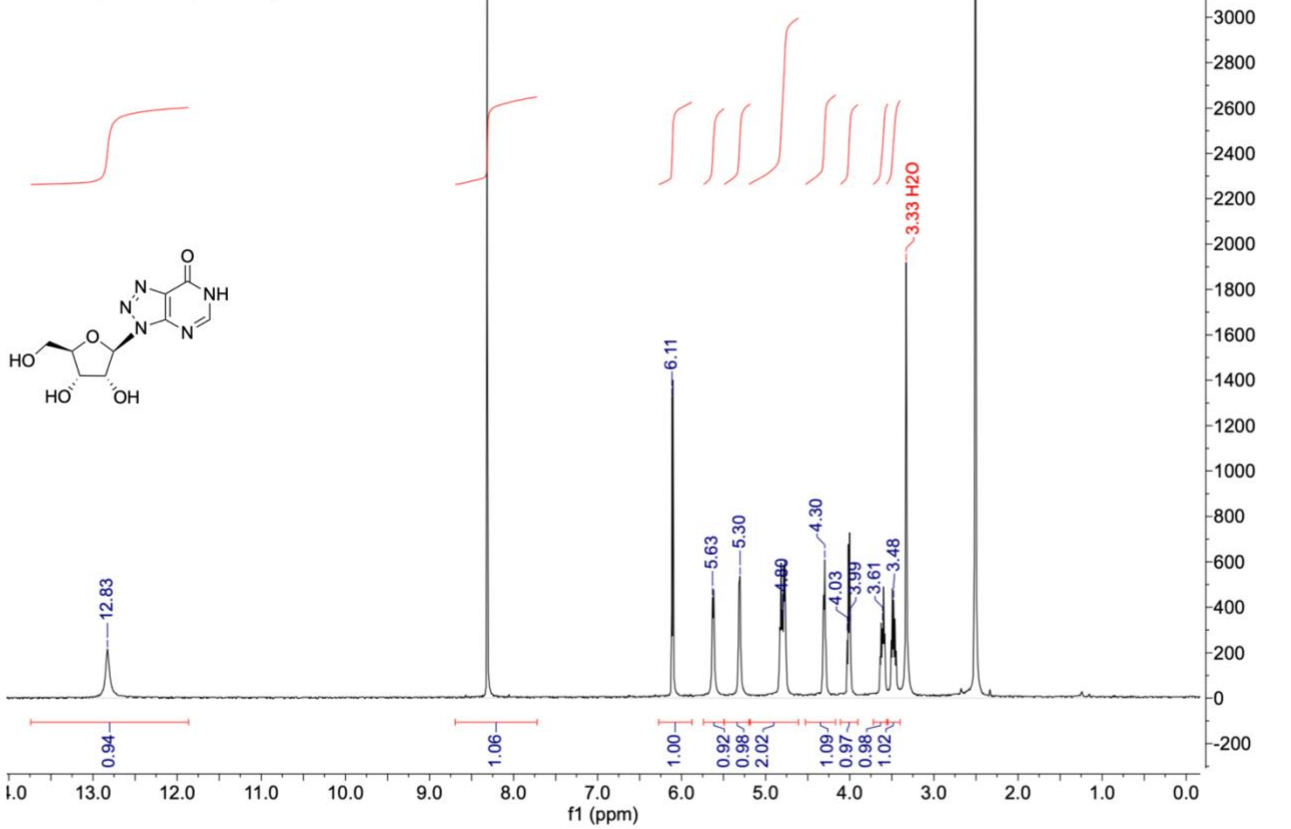

^{13}C NMR (76 MHz, DMSO) 8-azainosine

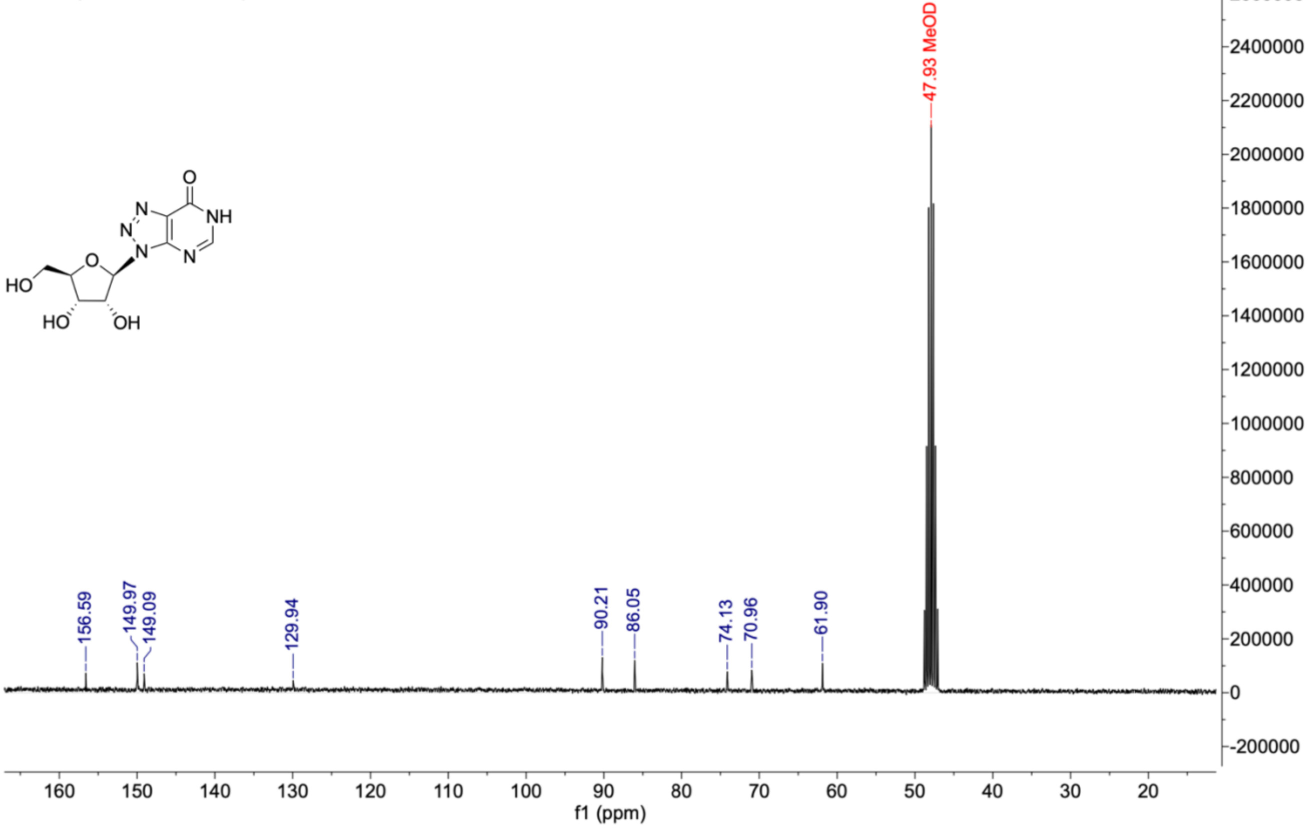

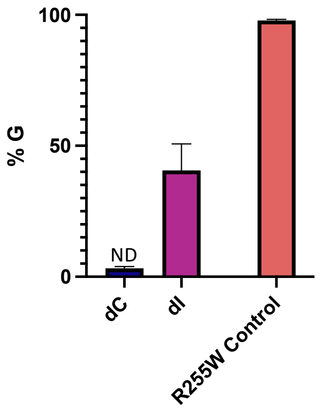

Figure S1. Effect of -1 analogs on cellular editing as measured by Sanger sequencing of RT-PCR amplicons [8]. Percent G measured by Sanger sequencing for the *MeCP2* R255X target site in HEK293T cells transfected with 30 nM of guide oligonucleotides bearing -1 dC or dI with overexpressed ADAR2. ND refers to G level below 5%. Value for the R255W control refer to the % G measured for an experiment using a reporter plasmid with an encoded tryptophan codon at *MeCP2* amino acid position 255 and represents the maximum possible % G.

References

1. Doherty, E. E.; Wilcox, X. E.; Van Sint Fiet, L.; Kemmel, C.; Turunen, J. J.; Klein, B.; Tantillo, D. J.; Fisher, A. J.; Beal, P. A. Rational Design of RNA Editing Guide Strands: Cytidine Analogs at the Orphan Position. *J Am Chem Soc* **2021**, *143* (18), 6865–6876. https://doi.org/10.1021/jacs.0c13319.
2. Doherty, E. E.; Karki, A.; Wilcox, X. E.; Mendoza, H. G.; Manjunath, A.; Matos, V. J.; Fisher, A. J.; Beal, P. A. ADAR Activation by Inducing a Syn Conformation at Guanosine Adjacent to an Editing Site. *Nucleic Acids Res* **2022**, *50* (19), 10857–10868. https://doi.org/10.1093/nar/gkac897.
3. Lee, A. H. F.; Kool, E. T. Novel Benzopyrimidines as Widened Analogues of DNA Bases. *Journal of Organic Chemistry* **2005**, *70* (1), 132–140. https://doi.org/10.1021/jo0483973
4. Vonrhein, C., C. Flensburg, P. Keller, A. Sharff, O. Smart, W. Paciorek, T. Womack, and G. Bricogne. Data processing and analysis with the autoPROC toolbox. *Acta Crystallogr D Biol Crystallogr* **2011**, *67*(Pt 4):293-302. https://doi.org/10.1107/S0907444911007773
5. Tickle, I.J., C. Flensburg, P. Keller, W. Paciorek, A. Sharff, C. Vonrhein, and G. Bricogne, *STARANISO*. **2018-2023**, Global Phasing Ltd.: Cambridge, United Kingdom.
6. Williams, C.J., J.J. Headd, N.W. Moriarty, M.G. Prisant, L.L. Videau, L.N. Deis, V. Verma, D.A. Keedy, B.J. Hintze, and V.B. Chen. MolProbity: More and better reference data for improved all-atom structure validation. *Protein Sci* **2018**, *27*:293-315. https://doi.org/10.1002/pro.3330
7. Véliz, E.A., Easterwood, L.M., Beal, P.A. (2003). Substrate Analogues for an RNA-Editing Adenosine Deaminse: Mechanistic Investigation and Inhibitor Design. *J Am Chem Soc* **2003,** *125*, 10867-10876. https://doi.org/10.1021/ja029742d
8. Quiroz, J. F. D.; Ojha, N.; Shayhidin, E. E.; De Silva, D.; Dabney, J.; Lancaster, A.; Coull, J.; Milstein, S.; Fraley, A. W.; Brown, C. R.; Rosenthal, J. J. C. Development of a Selection Assay for Small Guide RNAs That Drive Efficient Site-Directed RNA Editing. *Nucleic Acids Res* **2023**, 51 (7), E41. https://doi.org/10.1093/nar/gkad098
